# A Wide Range of Strategies to Cope with Healthcare Workers’ Vaccine Hesitancy in A North-Eastern Italian Region: Are They Enough?

**DOI:** 10.3390/healthcare9010004

**Published:** 2020-12-23

**Authors:** Laura Brunelli, Francesca Antinolfi, Francesca Malacarne, Roberto Cocconi, Silvio Brusaferro

**Affiliations:** 1 Department of Medicine, University of Udine, 33100 Udine, Italy; antinolfi.francesca@spes.uniud.it (F.A.); francesca.malacarne@asugi.sanita.fvg.it (F.M.); silvio.brusaferro@uniud.it (S.B.); 2 Quality and Accreditation Unit, Friuli Centrale Healthcare University Trust, 33100 Udine, Italy; roberto.cocconi@asufc.sanita.fvg.it

**Keywords:** healthcare workers, vaccine hesitancy, influenza, vaccine preventable disease, strategy

## Abstract

The recent pandemic reminded the world of the high risk of healthcare workers (HCWs) and patient contagiousness along with the healthcare services disruption related to nosocomial outbreaks. This study aims at describing vaccination campaigns within healthcare institutions of a North-Italian Region and comparing their effectiveness in term of vaccination coverage. In December 2019, we surveyed all healthcare institutions of Friuli Venezia Giulia Region throughout an email questionnaire with 15 questions investigating strategies adopted for the vaccination of HCWs against influenza and other vaccine-preventable diseases (VPDs), along with actions put in place in case of a VPD exposure. We found a strong heterogeneity in VPDs prevention and control policy and practice for HCWs, along with responsibility attribution ranging among different stakeholders. Strategies adopted to promote vaccination included a wide range of methods, but HCWs’ influenza vaccination coverage still ranged from 17.0 to 33.3%. Contact tracing after a VPD exposure did not always include medical residents and students and visitors/caregivers/extra personnel as possible contacts. Even if knowledge and complacency gaps among HCWs could be faced with education activities, more efforts should be done in identifying and implementing effective vaccination strategies, and mandatory vaccination for HCWs could be introduced to achieve host, herd, and healthcare immunity preventing possible hospital outbreaks.

## 1. Introduction

The recent severe acute respiratory syndrome coronavirus 2 (SARS-CoV-2) pandemic [1] once again highlighted the high risk of exposure to communicable diseases of healthcare workers (HCWs) and the threat posed in terms of patients’ contagiousness and healthcare services disruption by nosocomial outbreaks [2]. Patients with pre-symptomatic or even already clinically evident vaccine preventable diseases (VPDs) often seek advice from healthcare services, but the delayed disease recognition along with the late adoption of transmission prevention and control measures can cause hospital outbreaks [2]. Nosocomial transmission of a communicable disease depends on the disease basic reproduction number (R0) along with host and herd immunity, but also on HCWs vaccination/seroprevalence rates (healthcare immunity). Even if, on one hand, host and herd immunity can be fostered throughout vaccination campaigns conducted in the community, at-risk group of patients (e.g., immunocompromised, newborns) may just rely on herd immunity to be protected. On the other hand, much can be done as far as the nosocomial risk of acquiring VPDs by adopting prevention and control measures in healthcare institutions (e.g., personal protective equipment wearing, spatial division), increasing diagnosis preparedness, and reducing the possible reservoir of susceptible subjects represented by unvaccinated HCWs [3]. In fact, when available, HCW immunization has proven to be the most effective [2] and cost-saving means for nosocomial infection prevention and control [4,5]. Global and national data about HCWs vaccine coverage are not satisfying, as in Italy just 65% of HCWs are vaccinated for varicella, 80.5% for measles-mumps-rubella (MMR), and 93.7% for pertussis [6]; moreover, influenza vaccine coverage among HCWs is even worse [6,7] still being far from reaching desired levels. Even if strongly recommended by the scientific community [5,8] and not largely opposed by HCWs [9,10], as long as HCWs vaccination is not mandatory, great importance should be given to vaccination campaign rendering within healthcare organizations [7]. This study aims at describing HCWs vaccination campaign design within healthcare institutions of a North Italian Region (Friuli Venezia Giulia Region), comparing different strategies also considering healthcare institutions’ characteristics and influenza vaccination coverage for 2019–2020 season. 

## 2. Materials and Methods

In December 2019, we surveyed the Medical Directorate of the seven healthcare institutions of Friuli Venezia Giulia Region, which is a 1,215,220-inhabitant region located in the North-East of Italy, at both Slovenian and Austrian borders. We used a three-section questionnaire with 15 questions to investigate: (A) Strategies adopted for the vaccination of HCWs against influenza and (B) against other VPDs (measles, mumps, rubella, varicella, diphtheria, tetanus, pertussis, hepatitis B, polio), along with (C) actions to be put in place in case of an exposure to these communicable diseases within the hospital. Influenza was considered in a separate section due to its typical annual epidemic occurrence, which requires repeated vaccination campaigns and vaccine administrations. Section A asked about who is responsible for the influenza vaccination campaign, strategies adopted to promote vaccination (continuing medical education courses, campaign presentation event, email invitation, poster display, leaflet distribution, notice on healthcare institution website, social media, video display), location and time schedule of vaccination offer, existence and timeliness of vaccination adherence monitoring system, and actions taken in case of denial (denial form signing, mandatory mask wearing, temporal reallocation in a safer setting, other, none). Section B explored the same issues related to VPDs other than influenza, and, in addition, asked about single VPDs included in the prevention program, and methods used to assess immunity state. Section C explored actions implemented after the occurrence of a VPD case within the healthcare institution in general and specifically referred to the groups of susceptible and immune HCWs (susceptibility assessment, contact tracing, temporal reallocation, other); eventual extension of the contact tracing to outer personnel was also examined. Descriptive statistics were used for data analysis. Data describing the human resources of the healthcare institutions, such as the number of employees, and quota of HCWs, medical residents, and students among them, were also collected. Questionnaires were sent via email, accompanied by a brief introduction of the study purposes and methods. Healthcare institutions were assigned a code (1 to 7) to accomplish confidentiality. Residing population for each healthcare institution was obtained from available ISTAT (Italian National Institute of Statistics) data as referred to 1 January 2019. Data about influenza vaccination coverage of HCWs at the regional level were also collected. Results were consequently shared and discussed with involved healthcare institutions for-improvement purposes.

## 3. Results

We received 100% of questionnaires back; two of them from High Research Institutes, two from hub academic hospitals, and three from spoke hospitals. The characteristics of each healthcare institution in terms of type of institution, human resources, and referring general population are reported in Table 1.

### 3.1. Influenza

In five out of seven cases, the responsibility of the influenza vaccination campaign is attributed to the Medical Directorate of the healthcare institution, in most cases in collaboration with the Infection Control Team (3/7, 43%), the Public Health Department (3/7, 43%), or the Occupational Health Unit (4/7, 57%). The four most adopted strategies to promote vaccination against influenza are email invitation (7/7, 100%), poster display (6/7, 86%), notice on institutional website (6/7, 86%), leaflet distribution (5/7, 71%); other actions included educational meeting (3/7, 43%), promotion via social media (3/7, 43%), and personal reminders (1/7, 14%). Email promotion was sent to every employee and medical resident; in most cases (6/7, 86%) a specific communication was sent to all unit directors and coordinators, in addition to heads of residency programs in hub academic hospitals, but just in one case medical students were included. 

All healthcare institutions (5/7, 71%) made available a passive vaccination offer, by setting up a dedicated room with fixed opening time where each HCWs has to go to receive the influenza vaccination. Vaccination session booking was possible just in two hospitals; otherwise the access was free and upon availability. In five healthcare institutions (71%), an active offer was added by setting a timetable of on-site vaccination for each building (in one healthcare institution), floor (in two healthcare institutions), or unit (in one healthcare institution), while in three healthcare institutions (43%), direct distribution of vaccine kits to units was performed. All the above-mentioned strategies were considered active considering the effort made by the healthcare institution for facilitating HCWs vaccine uptake. Healthcare institutions dedicated one to five hours weekly to this activity, while two of them (29%) gave plenty of availability guaranteeing vaccination at any time during daily working hours. A final report of HCWs’ vaccination compliance was drafted in most cases (6/7, 86%), but data were made timely available in less cases, in particular being weekly (4/7, 57%) or monthly (1/7, 14%). 

In case of HCW denial to get influenza vaccination, no counteractions were taken, nor additional prevention measures prescribed. Vaccination coverage of HCWs addressed by influenza vaccination campaign ranged from 17.0% (healthcare institution 6) to 33.3% (healthcare institution 3), achieving a global regional level of 24.9%. Detailed characteristics of influenza vaccination campaign along with vaccination coverage are reported in Table 2.

### 3.2. Other VPDs

Surveilled VPDs for HCWs always included measles, mumps, rubella, and hepatitis B; differences among healthcare institutions were found regarding diphtheria, tetanus, pertussis, polio, and varicella surveillance. In most cases (6/7, 86%) the Occupational Health Unit oversees the surveillance on HCWs immunity for VPDs, in some cases in collaboration with the Public Health Department (3/7, 43%), the healthcare institution Medical Directorate (2/7, 29%) or the Infection Control Team (1/7; 14%). Some institutions (5/7, 71%) put in place actions to specifically improve HCWs’ vaccination adherence, but several methods were used to identify susceptible HCWs including paper and electronic vaccination records consult and serology evaluation. Vaccinations were always offered in a dedicated room and timetable, but in two healthcare institutions, the HCW was required to access public health services outside the hospital to be vaccinated. Booking was always available, but a HCWs dedicated session was given within just three healthcare institutions. In case of HCW denial to get any of these VPD vaccination, countermeasures were prescribed by only three healthcare institutions (43%) including mandatory mask wearing when on duty (2/7, 29%), reallocation in a less risky unit (1/7, 14%), or denial form signing (1/7, 14%).

### 3.3. Actions Implemented in Case of Exposure

Contact tracing is required by all healthcare institutions after the occurrence of a VPD case. Just four healthcare institutions (57%) included medical residents, students, and visitors/caregivers/extra personnel (e.g., technicians, cleaners) among surveilled categories. HCWs susceptibility is assessed via vaccination record consult or by performing serology. When a susceptible HCW is exposed, he/she is asked not to come to work (5/7, 71%) or to be temporarily reallocated to a safer setting (4/7, 57%) depending on the risk assessment. Three healthcare institutions required the exposed HCW to wear a mask when on duty. Serology is repeated by four healthcare institutions (57%) to assess seroconversion. Strategies adopted toward extra personnel in most healthcare institutions included communication to the Occupational Health Unit (5/7, 71%), but just in two of them the susceptibility evidence at time zero was required.

## 4. Discussion

We found a strong heterogeneity among healthcare institutions in education, promotion, and vaccination offer strategies adopted for VPDs prevention and control policies and practice among HCWs, along with responsibility attribution of all these activities, although being them part of a unique and quite little Region. This findings confirms what previously reported for another Italian Region, Sardinia [11]. 

Multiple strategies were adopted by healthcare institutions to foster influenza vaccination coverage, including covering education, promotion, and access to vaccination issues as suggested by Tognetto et al. [7]. Actions put in place tackled classical convenience and complacency issues [12], which were confirmed in recent studies to characterize HCWs’ attitudes and practices towards vaccines [5,8]. Campaign presentation event and Continuing Medical Education (CME) courses were organized to inform and educate HCWs about the importance of the vaccination against seasonal influenza. Influenza vaccination promotion was performed via email invitation and reminder, poster and video display, leaflet distribution, notice on healthcare institutions website display, and social media similarly to what already reported for other hospitals [7,11]. Access to influenza vaccination was made available by Friuli Venezia Giulia healthcare institutions by combining passive and active vaccination offer, as well as direct distribution of vaccine kits to units for on-site vaccination, according to what suggested by Haviari et al. [13]. Healthcare institutions who included this latter strategy and adopted a multiple approach [7] achieved the best regional results reaching a maximum of 33.3% in influenza vaccination coverage among HCWs. Maybe the results achieved in some settings also benefit from them being an academic center, given that the presence of residents directly involved in promoting and administering vaccination within the healthcare institution can help in having more people vaccinating colleagues as well as young HCWs promoting and supporting the vaccination campaign. Even though being a remarkable result in comparison with what recently reported for other hospitals in Rome (ranging from 4.23 to 12.97%) [7], this coverage is still far from the desired 75% set by the Italian Ministry of Health as the minimum goal to be reached [14]. As claimed by colleagues [8], despite all time-consuming and costly efforts implemented by healthcare institutions, they do not appear to be enough to achieve and maintain adequate vaccination rates against influenza among HCWs.

The frequent nosocomial transmission and the high risk for HCWs of acquiring VPDs [13] has not been accompanied by sufficient vaccine coverage at neither the local [15], national [6,16], nor international level [17,18]. The highlighted management heterogeneity for influenza vaccination was confirmed for the other VPDs even with the extension to the list of diseases under surveillance. Differently from what reported for influenza, no actual vaccination campaign with education, promotion, and access to vaccination actions was implemented. VPDs vaccination offer was mostly passive, sometimes requiring HCWs to move outside the hospital to get their vaccination. Therefore, we believe that much more can be done in terms of healthcare institutions policies and practices for counteracting HCWs convenience-related issues included in vaccine hesitancy [12]. There were a few countermeasures taken regarding vaccination denial by a HCWs; maybe the effective request for those refusing to get vaccinated to wear a mask while on duty should be reconsidered at least to prevent droplet transmission [13] and as a nudging strategy towards vaccination [19].

Globally, our results seem to confirm healthcare students being somehow overlooked in HCWs immunization programs [5], as in two healthcare institutions the Medical Directorates were not able to report the number of students accessing their institution and a suspicious “zero residents” was reported by another healthcare institution. Moreover, students, as well as visitors, caregivers, and extra personnel, are not always included among categories for contact tracing, possibly amplifying the risk of nosocomial transmission and thus posing a threat over the whole health system. 

At the European level, Slovenian and Italian HCWs resulted to be the most skeptical professionals with regards to vaccination [9]. The discussion of our results with the surveyed healthcare institutions allowed all participants to compare strategies adopted, while indirectly increasing awareness about HCWs’ vaccination crucial role. Even though strategies adopted by single healthcare institutions has led in some cases to promising results, the adoption of a regional approach based on the most efficient strategies could be the next step to be implemented for improving coverage among HCWs of all healthcare institutions. Nevertheless, the introduction of compulsory vaccination for HCWs [8] and the adoption of specific countermeasures in case of denial [20], as already reported by colleagues, should be considered.

This study presents some limits, including the unavailability of a validated questionnaire for surveying vaccination campaign characteristics and the limited geographical context surveyed. Moreover, a social desirability bias from respondents cannot be ruled out.

## 5. Conclusions

In conclusion, despite several strategies adopted, coverage remains insufficient. On one hand, existing gaps in knowledge and complacency among HCWs should be faced with education activities and more efforts should be done in identifying and implementing effective, coordinated and homogeneous vaccination strategies. On the other hand, maybe the time is ripe for a change and mandatory vaccination for healthcare workers could be introduced at a national level to achieve host, herd, and healthcare immunity preventing possible hospital outbreaks.

## Figures and Tables

**Table 1:** Main healthcare institution characteristics.

Healthcare Institution Code	Type of Institution	Total Employees	Healthcare Workers *	Medical Residents	Total Students	Inhabitants **
1	High Research Institute	699	467 (67%)	22 (5%)	82	-
2	High Research Institute	873	793 (91%)	50 (6%)	100	-
3	Hub academic hospital	4336	3992 (92%)	402 (10%)	718	250,928
4	Hub academic hospital	2116	2065 (98%)	Unknown	Unknown	234,493
5	Spoke hospital	2528	2403 (95%)	Unknown	Unknown	312,533
6	Spoke hospital	1215	1087 (89%)	12 (1%)	Unknown	168,060
7	Spoke hospital	2235	1820 (81%)	0	100	249,206

Unknown = data not available to the respondent * including medical residents and students, ** residing population for each healthcare institution, data from ISTAT referring to 1 January 2019.

**Table 2:** Strategies adopted for the 2019–2020 influenza vaccination campaign and outcomes.

Healthcare Institution Code	Target Population (*n*)	Strategies Adopted			Vaccination Coverage (%)
		Education	Promotion	Vaccination Offer	
1	467	Campaign presentation event	Email invitation, poster display, leaflet distribution, notice on healthcare institution website	Passive, active	24.4
2	793	-	Email invitation, poster display, leaflet distribution, reminder	Passive, active	30.9
3	3992	CME * course	Email invitation, poster display, notice on healthcare institution website	Passive, active, distribution of vaccine kits to units	33.3
4	2065	-	Email invitation, poster display, leaflet distribution, notice on healthcare institution website, social media	Passive, distribution of vaccine kits to units	18.5
5	2403	-	Email invitation, poster display, leaflet distribution, notice on healthcare institution website, social media, video display	Passive, active	23.5
6	1087	-	Email invitation notice on healthcare institution website	Passive, distribution of vaccine kits to units	29.4
7	1820	CME * course	Email invitation, poster display, leaflet distribution, notice on healthcare institution website, social media	Passive, active	17.0

* CME: Continuing medical education.
